# Sodium Butyrate Ameliorates Oxidative Stress-Induced Intestinal Epithelium Barrier Injury and Mitochondrial Damage through AMPK-Mitophagy Pathway

**DOI:** 10.1155/2022/3745135

**Published:** 2022-01-29

**Authors:** Xin Li, Chunchun Wang, Jiang Zhu, Qian Lin, Minjie Yu, Jiashu Wen, Jie Feng, Caihong Hu

**Affiliations:** ^1^College of Animal Science, Zhejiang University, Hangzhou 310058, China; ^2^Key Laboratory of Molecular Animal Nutrition (Zhejiang University), Ministry of Education, China; ^3^Key Laboratory of Animal Nutrition and Feed Science (Eastern of China), Ministry of Agriculture and Rural Affairs, China; ^4^Key Laboratory of Animal Feed and Nutrition of Zhejiang Province, China

## Abstract

Sodium butyrate has gained increasing attention for its vast beneficial effects. However, whether sodium butyrate could alleviate oxidative stress-induced intestinal dysfunction and mitochondrial damage of piglets and its underlying mechanism remains unclear. The present study used a hydrogen peroxide- (H_2_O_2_-) induced oxidative stress model to study whether sodium butyrate could alleviate oxidative stress, intestinal epithelium injury, and mitochondrial dysfunction of porcine intestinal epithelial cells (IPEC-J2) in AMPK-mitophagy-dependent pathway. The results indicated that sodium butyrate alleviated the H_2_O_2_-induced oxidative stress, decreased the level of reactive oxygen species (ROS), increased mitochondrial membrane potential (MMP), mitochondrial DNA (mtDNA), and mRNA expression of genes related to mitochondrial function, and inhibited the release of mitochondrial cytochrome c (Cyt c). Sodium butyrate reduced the protein expression of recombinant NLR family, pyrin domain-containing protein 3 (NLRP3) and fluorescein isothiocyanate dextran 4 kDa (FD4) permeability and increased transepithelial resistance (TER) and the protein expression of tight junction. Sodium butyrate increased the expression of light-chain-associated protein B (LC3B) and Beclin-1, reduced the expression of P62, and enhanced mitophagy. However, the use of AMPK inhibitor or mitophagy inhibitor weakened the protective effect of sodium butyrate on mitochondrial function and intestinal epithelium barrier function and suppressed the induction effect of sodium butyrate on mitophagy. In addition, we also found that after interference with AMPK*α*, the protective effect of sodium butyrate on IPEC-J2 cells treated with H_2_O_2_ was suppressed, indicating that AMPK*α* is necessary for sodium butyrate to exert its protective effect. In summary, these results revealed that sodium butyrate induced mitophagy by activating AMPK, thereby alleviating oxidative stress, intestinal epithelium barrier injury, and mitochondrial dysfunction induced by H_2_O_2_.

## 1. Introduction

The intestinal epithelium barrier is known as the important barrier to prevent the invasion of toxins or antigens [1]. Studies have found that oxidative stress is involved in the intestinal barrier impairment of piglets, which mainly refers to the imbalance of reactive oxygen species (ROS) and antioxidant system [2, 3]. Under oxidative stress, the overproduction of ROS can cause lipid peroxidation, protein, and DNA damage [4]. Multiple studies have shown that dietary sodium butyrate can protect the intestinal barrier of piglets by providing energy for intestinal epithelial cells, anti-inflammation, histone deacetylation, immune regulation [5–7], etc. The *in vivo* rat experiments and *in vitro* intestinal epithelial cell experiments have demonstrated that the protective effects of intestinal barrier function by sodium butyrate are related to its antioxidant capacity [8–10]. Since mitochondria are the main resource of ROS, the antioxidant effect of sodium butyrate may result from the targeting on mitochondria.

The gut needs a lot of energy to maintain homeostasis, which is mainly supplied by intracellular mitochondria [9, 10]. However, under oxidative stress, mitochondria are not only the main resource of ROS but also an important attack target of it [11]. Impaired mitochondria induced by oxidative stress can produce more than ten times more ROS than the normal mitochondria, which further aggravated mitochondrial damage [12]. In order to block this vicious cycle, the body will selectively remove damaged mitochondria via lysosome degradation, a process called mitophagy [13]. A recent study reported that sodium butyrate can induce mitophagy in Chinese hamster ovary cells [14]. We speculate that the antioxidant effect of sodium butyrate is related to decreasing mitochondrial ROS production and mitophagy.

Adenosine monophosphate-activated protein kinase (AMPK) is a key energy sensor, as well as an important oxidative stress sensor, which exerts an important role in mitochondrial function and mitophagy [15, 16]. AMPK*α* is the catalytic core of AMPK, and Toyama et al. found that AMPK is activated in cells under energy-deficient situation, as indicated by that AMPK promoted the further recruitment of multiple proteins related to mitochondrial division by phosphorylating the downstream protein mitochondrial fission factor (MFF), thereby causing mitochondrial division [17, 18], which links the energy receptor AMPK with mitochondrial division. It is well known that sodium butyrate is an important energy substance for intestinal epithelial cells and has the functions of alleviating oxidative stress and protecting the intestinal barrier [9, 19]. Mollica et al. have reported that feeding sodium butyrate to insulin-resistant obese mice can increase liver AMPK activity, reduce ROS production, and improve liver mitochondrial function [20]. However, whether sodium butyrate can alleviate oxidative stress and improve mitochondrial function through AMPK remains unclear. Further research is needed to study the mechanism through which sodium butyrate alleviates oxidative stress and improves intestinal barrier function.

Therefore, this experiment utilized an IPEC-J2 cell oxidative stress model by H_2_O_2_ to study the effects of sodium butyrate on intestinal barrier injury, mitochondrial function, mitophagy, and the underlying molecular mechanisms.

## 2. Material and Methods

### 2.1. Chemicals

The IPEC-J2 cell was a courtesy from Prof. Yin Yulong, Institute of Subtropical Agriculture, Chinese Academy of Sciences. H_2_O_2_ (Sinopharm Group, China), DMEM-F12 medium (Shanghai Yuanpei Biotechnology, China), and fluorescein isothiocyanate dextran 4 kDa (FD4) were obtained from Sigma-Aldrich (St. Louis, Missouri, USA). Trypsin (Beyotime Biotechnology, China), phosphate-buffered saline (PBS) (Bozan Biotechnology, China), penicillin-streptomycin (Solabao Biotechnology, China), fetal bovine serum (Gemini, Australia), CCK-8 kit (Beyotime Biotechnology, China), MitoSpy™ Red CMXRos (Biolegend, USA), immunofluorescence fixative (Sevier Biotechnology, China), Triton-X 100 (Sigma-Aldrich, St. Louis, MO, USA), Glycine (Sinopharm Group, China), DAPI (Beyotime Biotechnology, China), Goat Anti-Mouse IgG Dylight 594 (Earthox, USA), Goat Anti-Mouse IgG Dylight 488 (Earthox, USA), mitochondrial division inhibitor (Mdivi-1) (Selleck, USA), AMPK inhibitor (Compound C, CC) (Selleck, USA), Lipofectamine RNAiMAX, and Lipofectamine 2000 were obtained from Invitrogen (Invitrogen, USA).

### 2.2. Experimental Design and Cell Culture

IPEC-J2 cells were cultured in DMEM-F12 complete medium (10% fetal bovine serum and 1% antibiotics (penicillin and streptomycin)). The culture conditions are 37°C, 5% CO_2_, and 95% humidity in a carbon dioxide incubator, and the medium is changed every 2 days. The IPEC-J2 cell oxidative stress model is constructed by 600 *μ*mol/L H_2_O_2_ for 8 hours. This study is divided into three experiments. Experiment 1: control group (control), sodium butyrate group (NaB), oxidative stress group (H_2_O_2_), and sodium butyrate+oxidative stress group (NaB+H_2_O_2_); 600 *μ*mol/L H_2_O_2_ was treated with cells in the H_2_O_2_ group and NaB+H_2_O_2_ group for 8 hours. The cells in the control group and NaB group were added with the same amount of PBS. Experiment 2: control group (control), oxidative stress group (H_2_O_2_), sodium butyrate+oxidative stress group (NaB+H_2_O_2_), sodium butyrate+oxidative stress+mitophagy inhibitor group (NaB+H_2_O_2_+Mdivi-1), and sodium butyrate+oxidative stress+AMPK signal pathway inhibitor group (NaB+H_2_O_2_+Compound C); 1 hour before adding H_2_O_2_ or PBS, mitophagy inhibitor (Mdivi-1, 1 *μ*mol/L) or AMPK signaling pathway inhibitor (Compound C, 10 *μ*mol/L) was added into cells. Experiment 3: the experiment is divided into six groups: control group (control+siAMPK*α*), oxidative stress group (H_2_O_2_), oxidative stress+interference AMPK*α* (H_2_O_2_+siAMPK*α*), sodium butyrate+oxidative stress group (NaB+H_2_O_2_), and sodium butyrate+oxidative stress+interference AMPK*α* group (NaB+H_2_O_2_+siAMPK*α*). The cells in control, H_2_O_2_, and NaB+H_2_O_2_ groups were transfected with siControl. And the cells in control+siAMPK*α*, H_2_O_2_+siAMPK*α*, and NaB+H_2_O_2_+siAMPK*α* groups were transfected with siAMPK*α*.

### 2.3. Cell Viability Experiment

IPEC-J2 cells in logarithmic phase were seeded in a 96-well plate, 10 *μ*l of CCK-8 solution was added to each well, incubated at 37°C for 4 h, and the absorbance at 450 nm was measured with a microplate reader (FLx800, Bio-Tek Instruments Inc., Winooski, USA). The cell viability is normalized based on the absorbance of cells in the control group.

### 2.4. Transepithelial Electrical Resistance (TER) and FD4 Flux of IPEC-J2 Cells in the Transwell System

According to the previous study, transwell (Corning, NT, USA) is premoistened with DMEM-F12 medium in the incubator for more than 30 minutes before use [21]. After trypsinization of the cells, stop the reaction with fresh medium, centrifuge at 1500 rpm for 5 min, discard the solution and add a certain volume of fresh medium, adjust the cell concentration to 1 × 10^6^ cells/ml, add 150 *μ*l cells to the upper chamber suspension, add 1.5 ml of fresh medium to the lower chamber, and place it in a carbon dioxide incubator for culture. Change the fluid every 2 days. According to the previous study, TER was measured by the Millicell-ERS resistance system (Millipore; Bedford, MA) at three different points in each transwell, and the average of the three measured values was actual TER [22]. The TER value measured without inoculation of the cell culture is the blank TER. The cell monolayer TER = (measured TER − Blank TER) × transwell effective membrane area and expressed in (*Ω*·cm^2^). FD4 (final concentration 1 mg/ml) was added to the upper chamber of transwell, and 50 *μ*l (supplement with the same volume of fresh medium after sampling) was sampled in the lower chamber at 24 hours and 48 hours, to determine the concentration of FD4 and calculate the permeability of the intestinal epithelium barrier FD4 by a fluorescence microplate reader (FLx800, Bio-Tek Instruments Inc., Winooski, USA).

### 2.5. Determination of Cellular Antioxidant Capacity and IL-1*β*

After cell treatment, add cell lysate, lyse on ice for 10 min, centrifuge at 4°C for 10 min (12000 rpm/min), and take the supernatant for testing. According to the ELISA kit instructions (Nanjing Jiancheng Bioengineering Institute, Jiangsu, China), the activity of superoxide dismutase (SOD) and GSH (glutathione reductase) antioxidant enzymes, the MDA (malondialdehyde) content, and IL-1*β* content were measured for each sample.

### 2.6. Determination of Cellular ROS Level

Flow cytometry was conducted to determine the cellular ROS level by a ROS-sensitive fluorescence indicator 2′,7′-dichlorofluorescein diacetate (DCFH-DA; Sigma-Aldrich) [23]. In brief, the cells in the 2 ml centrifuge were incubated with 1 ml serum-free diluted DCFH-DA (1 : 1000) at 37°C for 30 min in the dark. After washed twice with PBS and resuspended, flow cytometry (BD, USA) was used to detect the cellular ROS. The data was calculated by FlowJo software 10.4 (V 7.6.1).

### 2.7. Measurement of Mitochondrial Membrane Potential (MMP)

The mitochondrial membrane potential was measured by mitochondrial membrane potential assay kit (Beyotime Biotechnology, China) according to the previous study [24]. JC-1 monomers (green) can form aggregates (red fluorescence) in the mitochondria with high △*Ψ*m, which cannot form aggregates in the mitochondria with low △*Ψ*m. Briefly, the cells were incubated with JC-1 working solution 37°C for 25 minutes. After washed for three times with PBS, flow cytometry (BD, USA) or a confocal laser microscope (FV1000, Olympus, Japan) was used to detect mitochondrial membrane potential (MMP).

### 2.8. mRNA Expression Level Analysis by Quantitative Real-Time PCR (RT-qPCR)

The total RNA extraction was conducted by Trizol according to the manufacturer's instruction (Takara, Japan). After detecting RNA concentration and purity, it is used for cDNA synthesis (Vazyme, Nanjing, China) and then for RT-qPCR according to the manufacturer's instructions (qPCR, master mix, Vazyme, Nanjing, China) as previously described [25]. The primer sequences are shown in supplementary Table 1. Glyceraldehyde-3-phosphate dehydrogenase (GAPDH) was used as an internal reference gene to calculate the relative expression of each gene, and the data was normalized using the 2^-*ΔΔ*Ct^ method.

### 2.9. Western Blot Experiment

The western blot experiment was conducted according to the previous study [26]. Total protein extraction and quantification were performed through the RIPA lysis buffer and BCA protein Assay Kit (Beyotime Biotechnology, Shanghai, China). Proteins from the jejunum mucosa were run and isolated through SDS-PAGE. Then, proteins were transferred to a polyvinylidene difluoride membrane. After blocking with 5% defat milk powder at room temperature for one hour, blots were incubated with specific primary antibodies overnight and then with horseradish peroxidase- (HRP-) conjugated secondary antibodies. BeyoECL Moon kit (Beyotime Biotechnology, China) was used to visualize the protein blot. The primary antibodies including *β*-actin, PINK1, Parkin, Claudin-1, Occludin, ZO-1, LC3B, Beclin-1, and P62 were purchased from Santa Cruz Biotechnology. Cytochrome c, NLRP3, Caspase-1, IL-1*β*, HRP-conjugated anti-rabbit IgG, and HRP-conjugated anti-mouse IgG antibodies were purchased from HUABIO (Hangzhou, China).

### 2.10. Ultrastructure of IPEC-J2 Cell Mitochondria

Briefly, the IPEC-J2 cells were fixed in 2.5% glutaraldehyde at 4°C overnight and then postfixed with 1% OsO4. After dehydration (gradient concentration of ethanol), infiltration (Spurr resin), embedding, ultrathin sectioning (LEICA EM UC7), and staining (uranyl acetate and alkaline lead citrate for 5 to 10 minutes, respectively), the samples were observed by transmission electron microscope (Hitachi Model H-7650, Tokyo, Japan).

### 2.11. Mitochondria Mark by MitoSpy™ Red CMXRos

MitoSpy™ Mitochondrial Localization Probe (Biolegend, USA) is a cell-permeable fluorescent chemical reagent used to label mitochondria in living cells. According to the manufacturer's instruction, we centrifuge the lyophilized reagent in the centrifuge tube to ensure that the reagent is at the bottom of the vial, add 94 *μ*l DMSO to each tube, and reconstitute MitoSpy™ Red CMXRos to a concentration of 1 mmol/L with DMSO. The stock solution is diluted in serum-free medium to make MitoSpy™ Red CMXRos working solution. After the cell treatment is over, wash once with warm 1X PBS. Add the diluted MitoSpy™ Red CMXRos solution to living cells and place them in an incubator at 37°C for 20-30 min. Wash the cells twice with warm 1X PBS or medium. Fix the cells with immunofluorescence fixative (4% paraformaldehyde) for 10-20 minutes at room temperature. Wash the cells twice with 1X PBS for subsequent experiments.

### 2.12. Immunofluorescence Analysis

The IPEC-J2 cells were seeded in a laser confocal culture dish. After the indicated treatment, the cells were washed twice with precooled PBS, and go through the steps of fixation, permeabilization (Tritox X-100, Beyotime Biotechnology, Shanghai, China), blocking, primary antibody incubation (Claudin-1, LC3B, and PINK1), secondary antibody incubation (Dylight-conjugated, Earthox, USA), nuclear staining (DAPI), and sealing. The samples were observed and photographed under a confocal laser microscope (FV1000, Olympus, Japan).

### 2.13. siRNA and Cell Transfection

IPEC-J2 cells were seeded into 6-well plates to grow about 80% confluent. The next day, individual targeted siRNA and plasmid were mixed with Lipofectamine RNAiMAX or Lipofectamine 2000 (Invitrogen, USA). The siRNA sequence is as follows: siAMPK*α* (5′-GCT GCACCAGAAGTAATTTTT-3′) and siControl (5′-UUCUCCGAACGUGUCACG UTT-3′). The RNAiMAX/siRNA mixture was added to IPEC-J2 cells in antibiotic-free medium and cultured for 4-6 h. Medium containing siRNA was refreshed with the general medium. Sodium butyrate and H_2_O_2_ were added to the indicated group according to the experimental design. Then, the samples were collected for subsequent experiments.

### 2.14. Statistical Analysis

The experimental data was statistically analyzed using the SPSS 20.0 software. One-way ANOVA was used for test analysis. Turkey method was used for multiple comparisons, and the GraphPad Prism 7.01 software was used for graphing. *P* < 0.05 indicates significant difference.

## 3. Results

### 3.1. Effect of Sodium Butyrate on the Oxidative Stress of IPEC-J2 Cells Treated by H_2_O_2_

**Figure 1:**
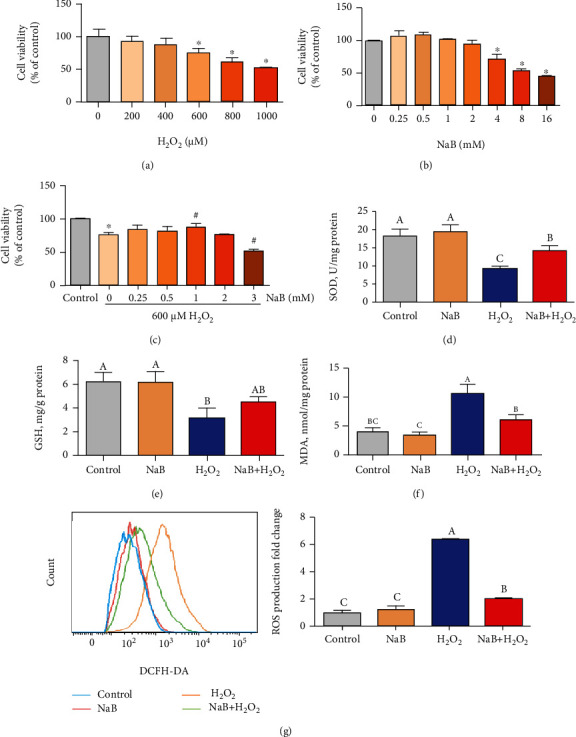
Effects of sodium butyrate (NaB) on the oxidative stress of porcine intestinal epithelial cells (IPEC-J2) treated by hydrogen peroxide (H2O2). (a–c) The effect of different concentrations of H2O2 and NaB on cell viability. (d–f) Cell superoxide dismutase (SOD), glutathione reductase (GSH) activity, and malondialdehyde (MDA) level. (g) Flow cytometry to quantify the reactive oxygen species (ROS) level of IPEC-J2 cells. Different superscript letters within the same row mean significant difference (*P* < 0.05). ∗ and # mean significant differences (*P* < 0.05) with the control group.

The IPEC-J2 cells were treated with different concentrations of H_2_O_2_ (200 *μ*mol/L, 400 *μ*mol/L, 600 *μ*mol/L, 800 *μ*mol/L, and 1000 *μ*mol/L) for 8 hours, and the cell viability was determined. As shown in Figure 1(a), it was found that H_2_O_2_ of 600, 800, and 1000 *μ*mol/L significantly reduced cell viability (*P* < 0.05). Cells were treated with 0.25, 0.5, 1, 2, 4, 8, and 16 *μ*mol/L sodium butyrate for 24 hours, and it was found that 0.25-2 mmol/L sodium butyrate had no significant effect on cell viability, and more than 4 mmol/L would significantly inhibit cell growth (Figure 1(b)). In addition, the cells were treated with 0.25, 0.5, 1, 2, and 4 mmol//L sodium butyrate, and it was found that 1 mmol/L sodium butyrate could effectively inhibit the decrease in cell viability induced by H_2_O_2_ (*P* < 0.05) (Figure 1(c)). Therefore, the follow-up experiments used 600 *μ*mol/L H_2_O_2_-induced oxidative stress model and 1 mmol/L sodium butyrate pretreatment. As shown in Figures 1(d)–1(f), compared with the control group, H_2_O_2_ treatment significantly reduced the SOD and GSH activities of IPEC-J2 cells (*P* < 0.05) and increased the MDA level (*P* < 0.05); meanwhile, compared with the H_2_O_2_ group, sodium butyrate significantly inhibited the decreased activity of SOD and GHS induced by H_2_O_2_, alleviated the increase of MDA level (*P* < 0.05). Similarly, flow cytometry data showed that, in comparison with the control group, sodium butyrate pretreatment significantly inhibited the increase in ROS levels induced by H_2_O_2_ (*P* < 0.05) (Figure 1(g)).

### 3.2. Effect of Sodium Butyrate on the Mitochondrial Function of IPEC-J2 Cells Treated with H_2_O_2_

**Figure 2:**
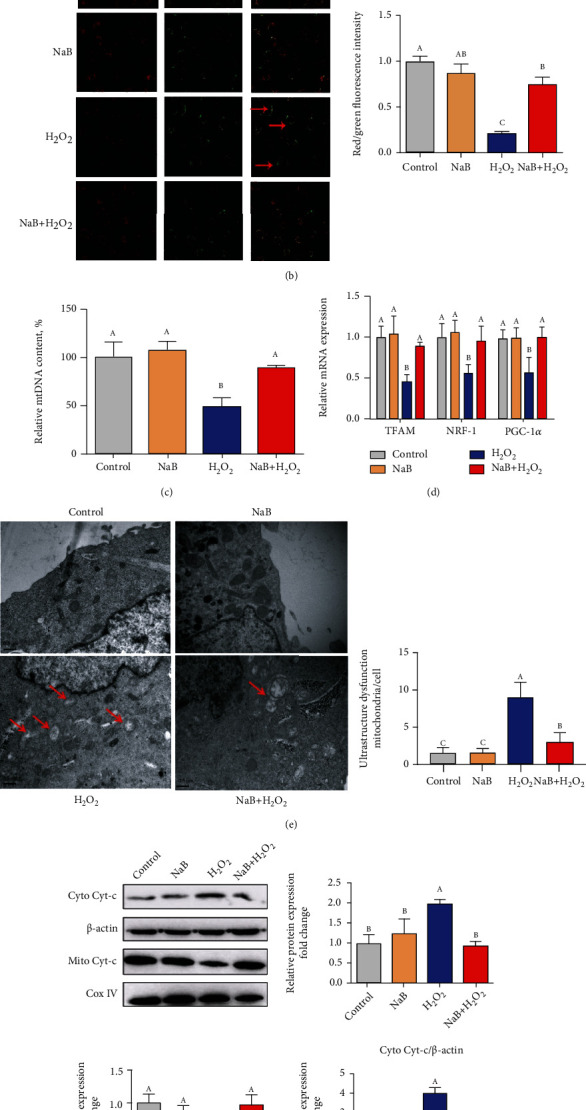
Effects of sodium butyrate (NaB) on the mitochondrial function of porcine intestinal epithelial cells (IPEC-J2) treated with H2O2. (a) Flow cytometry to detect changes in cell mitochondrial membrane potential. (b) The ratio of red to green of mitochondria JC-1 under a laser confocal microscope and quantification results. (c, d) Mitochondrial DNA (mtDNA) content and mitochondrial transcription factor A (TFAM), nuclear respiratory factor-1 (NRF-1), and peroxisomal proliferator-activated receptor-g coactivator-1*α* (PGC-1*α*) gene expression. (e) Ultrastructure of mitochondria under a transmission electron microscope (TEM). (f) Protein expression and quantification of cytochrome c. Different superscript letters within the same row mean significant difference (*P* < 0.05).

Flow cytometry data showed that compared with the control group, H_2_O_2_ significantly increased the proportion of depolarized cells (*P* < 0.05), while sodium butyrate pretreatment reversed the increase in the proportion of depolarized cells induced by H_2_O_2_ (*P* < 0.05) (Figure 2(a)). In addition, under laser confocal microscope, we found that compared with the control group, H_2_O_2_ reduced the ratio of JC-1 aggregates to monomer (*P* < 0.05), indicating that the mitochondrial membrane potential was reduced, and the sodium butyrate treatment alleviated the decrease of cell mitochondrial membrane potential induced by H_2_O_2_ (*P* < 0.05) (Figure 2(b)). By detecting the amount of mitochondrial DNA (mtDNA) (Figure 2(c)) and the mRNA expression level of mitochondrial function-related genes (Figure 2(d)), it was found that compared with the control group, H_2_O_2_ treatment significantly reduced the amount of mtDNA and the mRNA expression of mitochondrial function-related gene mitochondrial transcription factor A (TFAM), nuclear respiratory factor-1 (NRF-1), and peroxisomal proliferator-activated receptor-g coactivator-1*α* (PGC-1*α*) (*P* < 0.05); sodium butyrate significantly alleviated the reduction of mtDNA and mRNA level of mitochondrial function-related genes induced by H_2_O_2_ (*P* < 0.05). Similarly, under the transmission electron microscope, it was observed (Figure 2(e)) that the cells in the H_2_O_2_ group showed obvious mitochondrial swelling, mitochondrial crista breakage, and mitochondrial vacuolization; sodium butyrate significantly improved the ultrastructure of mitochondria. As shown in Figure 2(f), compared with the control group, H_2_O_2_ increased the level of cytochrome c (Cyt c) in the cytoplasm (*P* < 0.05) and decreased the level of Cyt c in the mitochondria (*P* < 0.05), suggesting that H_2_O_2_ increased the permeability of the inner and outer mitochondrial membranes and resulted in the release of mitochondrial Cyt c into the cytoplasm, indicating that the mitochondrial function is damaged; compared with the H_2_O_2_ group, sodium butyrate alleviated the release of mitochondrial Cyt c caused by H_2_O_2_ (*P* < 0.05), indicating that sodium butyrate treatment can help improve mitochondrial function under oxidative stress.

### 3.3. Effect of Sodium Butyrate on Inflammasome and Inflammatory Factors of IPEC-J2 Cells Treated with H_2_O_2_

**Figure 3:**
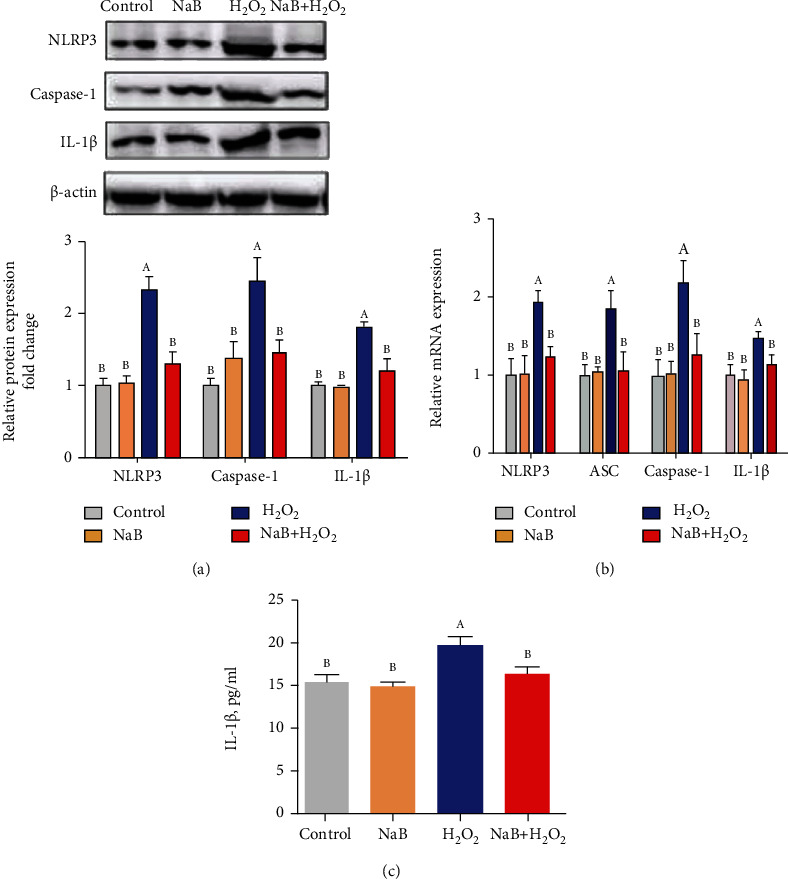
Effects of sodium butyrate (NaB) on H2O2 treatment of porcine intestinal epithelial cell (IPEC-J2) inflammasome and inflammatory factors. (a) Protein expression and quantification of recombinant NLR family, pyrin domain-containing protein 3 (NLRP3), Caspase-1, and IL-1*β*. (b) Relative gene expression of NLRP3, Caspase-1, ASC, and IL-1*β*. (c) The content of IL-1*β* in IPEC-J2 cells. Different superscript letters within the same row mean significant difference (*P* < 0.05).

As shown in Figures 3(a)–3(c), compared with the control group, H_2_O_2_ significantly increased the mRNA level and protein level of NLRP3, Caspase-1, and IL-1*β* in IPEC-J2 cells (*P* < 0.05) and increased the content of IL-1*β* (*P* < 0.05); compared with the H_2_O_2_ group, sodium butyrate significantly reduced the mRNA level and protein expression of NLRP3, Caspase-1, and IL-1*β* (*P* < 0.05) and reduced the content of IL-1*β* (*P* < 0.05).

### 3.4. Effect of Sodium Butyrate on the Intestinal Epithelium Barrier Function of IPEC-J2 Cells Treated with H_2_O_2_

**Figure 4:**
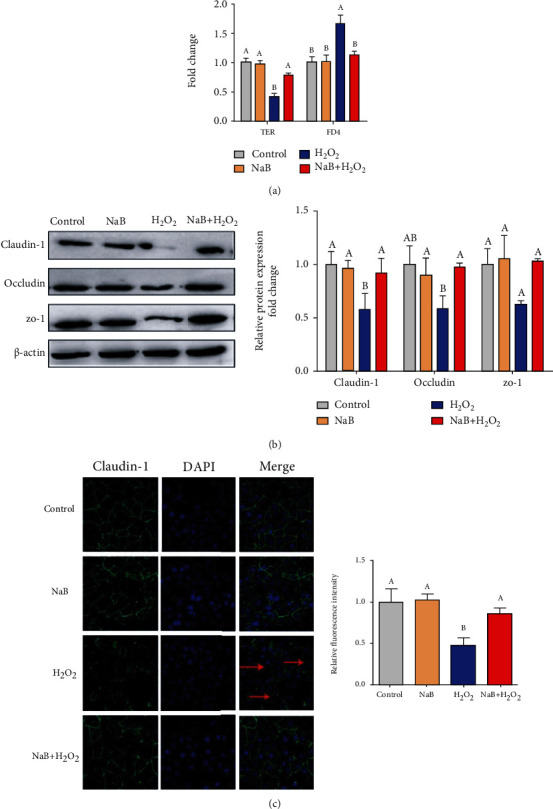
Effects of sodium butyrate (NaB) on the barrier function of porcine intestinal epithelial cells (IPEC-J2) treated with hydrogen peroxide (H2O2). (a) Intestinal epithelial transepithelial resistance (TER) and fluorescein isothiocyanate dextran 4 kDa (FD4) permeability. (b) Protein expression and quantification of Claudin-1, Occludin, and ZO-1. (c) The distribution and quantification of Claudin-1 protein under a confocal microscope. Different superscript letters within the same row mean significant difference (*P* < 0.05).

As shown in Figure 4(a), compared with the control group, H_2_O_2_ significantly reduced TER (*P* < 0.05) and increased the flux of FD4 (*P* < 0.05). Compared with the H_2_O_2_ group, sodium butyrate pretreatment significantly alleviated the H_2_O_2_-induced decrease in TER and the increase in FD4 flux (*P* < 0.05). In comparison with the control group, H_2_O_2_ significantly reduced the expression of Claudin-1, Occludin, and ZO-1 of IPEC-J2 cells (*P* < 0.05); compared with the H_2_O_2_ group, sodium butyrate pretreatment significantly increased the protein expression of Claudin-1, Occludin, and ZO-1 in IPEC-J2 cells (*P* < 0.05) (Figure 4(b)). As shown in Figure 4(c), the Claudin-1 protein of IPEC-J2 cells in the control group is mainly distributed near the cell membrane and forms a continuous ring around the cell membrane; but compared with the control group, the H_2_O_2_ treatment destroyed the normal distribution and expression of Claudin-1 (*P* < 0.05). The sodium butyrate pretreatment prevented the disorder of Claudin-1 distribution at the cell boundary and the decrease of protein expression caused by H_2_O_2_ (*P* < 0.05).

### 3.5. Effect of Sodium Butyrate on Mitophagy of IPEC-J2 Cells Treated with H_2_O_2_

**Figure 5:**
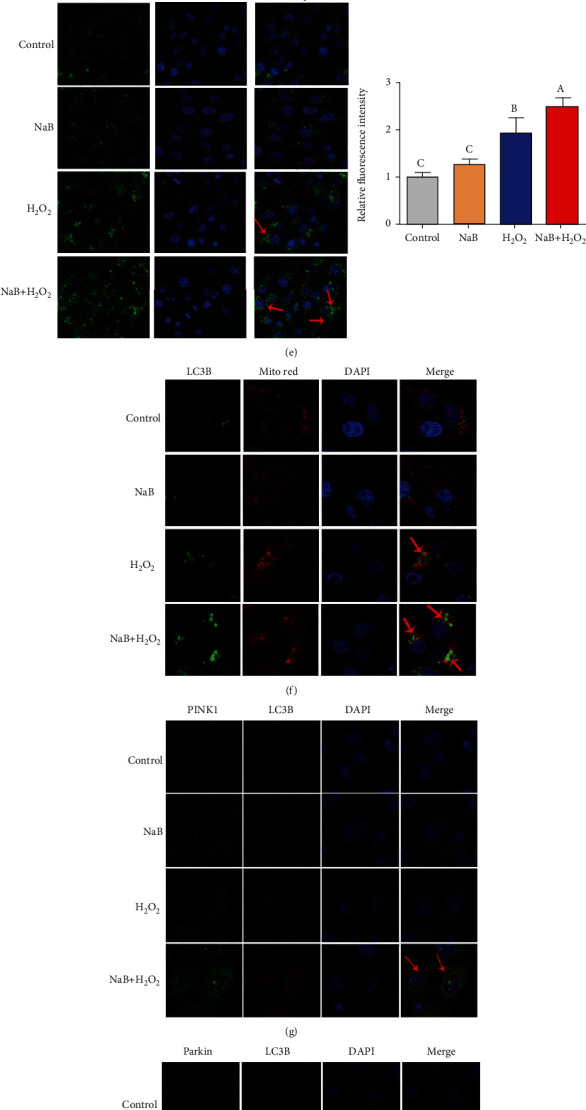
Effects of sodium butyrate (NaB) on mitophagy of porcine intestinal epithelial cells (IPEC-J2) treated with H2O2. (a, b) mRNA expression level of autophagy-related genes. (c, d) Western blot of autophagy-related protein expression and quantification. (e) The protein expression and quantification of light-chain-associated protein B (LC3B) under a laser confocal microscope. (f) Colocalization of mitochondrial Mito Red and autophagy protein LC3B under a laser confocal microscope. (g) Colocalization of mitophagy proteins PINK1 and LC3B under a laser confocal microscope. (h) Colocalization of mitophagy proteins Parkin and LC3B under a laser confocal microscope. (i) Mitophagy vesicles under transmission electron microscope. Different superscript letters within the same row mean significant difference (*P* < 0.05).

As shown in Figures 5(a)–5(d), compared with the control group, H_2_O_2_ significantly increased the mRNA expression of LC3B, Beclin-1, ATG 5, PINK1, and Parkin of IPEC-J2 cells and reduced the mRNA level of P62 (*P* < 0.05). Compared with the H_2_O_2_ group, sodium butyrate pretreatment further increased its mRNA level (*P* < 0.05) and reduced the mRNA expression of P62 (*P* < 0.05). Similarly, compared with the H_2_O_2_ group, sodium butyrate pretreatment further increased the protein expression of PINK1, Parkin, LC3B, and Beclin-1 (*P* < 0.05) and decreased the expression of P62 (*P* < 0.05). According to the immunofluorescence results (Figure 5(e)), we found that compared with the control group, H_2_O_2_ significantly increased the fluorescence intensity of LC3B of IPEC-J2 cells (*P* < 0.05); compared with the H_2_O_2_ group, sodium butyrate pretreatment further increased LC3B fluorescence intensity (*P* < 0.05). As shown in Figure 5(f), the results of confocal microscopy showed that under oxidative stress, sodium butyrate promotes the colocalization of LC3B and mitochondria and also increases the colocalization of PINK1, Parkin, and LC3B (Figures 5(g) and 5(h)). Similarly, according to the transmission electron microscope (TEM) results, we also observed the increase of mitophagy vesicles under the cotreatment of sodium butyrate and H_2_O_2_ (Figure 5(i)). These results indicate that sodium butyrate promotes mitophagy in H_2_O_2_-treated IPEC-J2 cells.

### 3.6. The Effect of Sodium Butyrate on Oxidative Stress, Intestinal Epithelium Barrier, and Mitophagy of IPEC-J2 Cells after Inhibition of Mitophagy or AMPK*α*

**Figure 6:**
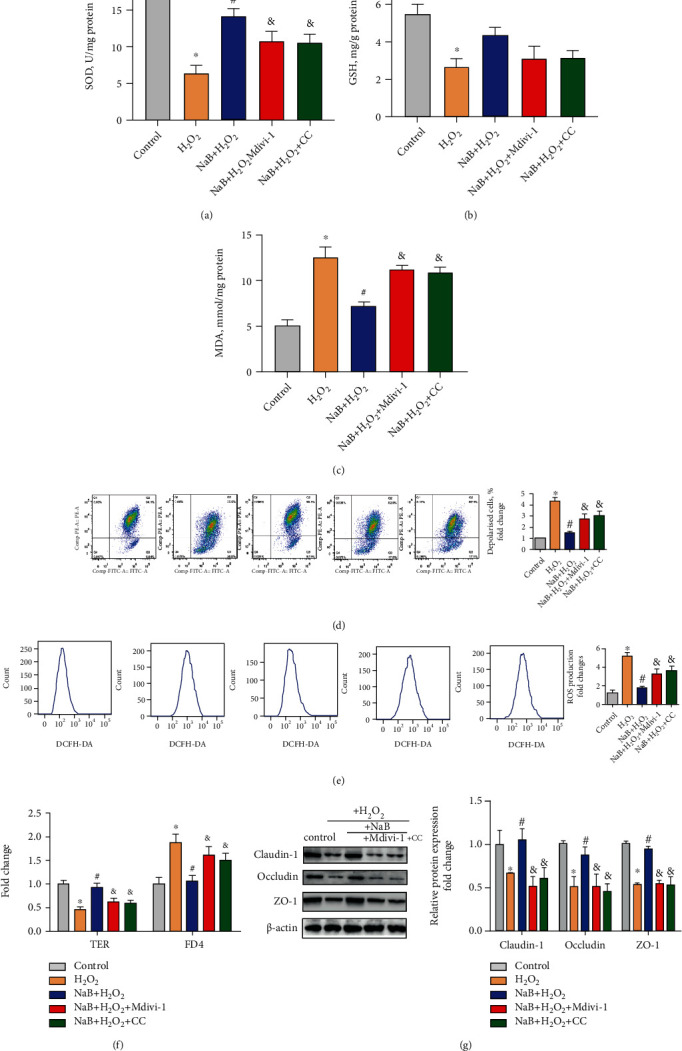
Effects of sodium butyrate (NaB) on oxidative stress, intestinal epithelium barrier, and mitophagy of porcine intestinal epithelial cells (IPEC-J2) after inhibiting mitophagy or AMPK. (a–c) Superoxide dismutase (SOD), glutathione reductase (GSH) activity, and malondialdehyde (MDA) content of IPEC-J2 treated with Mdivi-1 or Compound C (CC). (d) Cellular mitochondrial membrane potential quantification by flow cytometry. (e) Cellular reactive oxygen species (ROS) level quantification by flow cytometry. (f) Intestinal epithelial transepithelial resistance (TER) and fluorescein isothiocyanate dextran 4 kDa (FD4) permeability. (g) Protein expression and quantification of tight junction Claudin-1, Occludin, and ZO-1. ∗ indicates a significant difference compared with the control group (*P* < 0.05); # indicates a significant difference compared with the H2O2 group (*P* < 0.05); & indicates a significant difference compared with the NaB+H2O2 group (*P* < 0.05).

As shown in Figures 6(a)–6(c), in comparison with the H_2_O_2_ group, sodium butyrate increased the SOD activity (*P* < 0.05) and decreased the MDA level (*P* < 0.05). Compared with the NaB+H_2_O_2_ group, Mdivi-1 or Compound C treatment increased the MDA level (*P* < 0.05) and inhibited the SOD activity (*P* < 0.05). These results indicate that inhibition of mitophagy and AMPK*α* weakens the antioxidant effect of sodium butyrate. As shown in Figure 6(d), compared with the NaB+H_2_O_2_ group, Mdivi-1 and Compound C treatments significantly increased (*P* < 0.05) the percentage of depolarized cells (reflecting the decrease in mitochondrial membrane potential). Compared with the NaB+H_2_O_2_ group, Mdivi-1 and Compound C treatments significantly increased the ROS levels of IPEC-J2 cells (*P* < 0.05) (Figure 6(e)). These results indicate that inhibition of mitophagy or AMPK*α* weakens the protective effects of sodium butyrate on mitochondria under oxidative stress. As shown in Figures 6(f) and 6(g), compared with the NaB+H_2_O_2_ group, Mdivi-1 or Compound C treatment significantly reduced the TER (*P* < 0.05) and increased FD4 (*P* < 0.05). Mdivi-1 or Compound C treatment also reduced the expression of tight junction proteins Claudin-1, Occludin, and ZO-1 (*P* < 0.05). The data indicated that inhibition of mitophagy or AMPK*α* weakened the protective effects of sodium butyrate on intestinal epithelium barrier function under oxidative stress.

### 3.7. The Effect of Sodium Butyrate on the Mitophagy of IPEC-J2 Cells after Inhibiting Mitophagy or AMPK*α*

**Figure 7:**
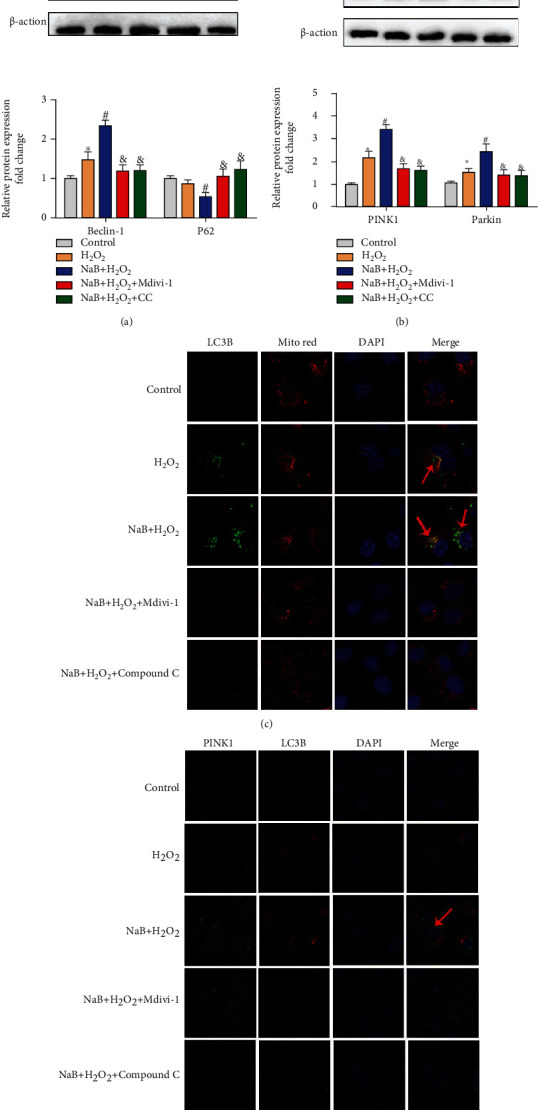
Effects of sodium butyrate (NaB) on mitophagy of porcine intestinal epithelial cells (IEPC-J2) after inhibition of mitophagy or AMPK. (a, b) Protein expression and quantification of autophagy protein. (c) Colocalization of mitochondria and light-chain-associated protein B (LC3B) under a laser confocal microscope. (d) Colocalization of mitophagy proteins PINK1 and LC3B under a laser confocal microscope. ∗ indicates a significant difference compared with the control group (*P* < 0.05); # indicates a significant difference compared with the H2O2 group (*P* < 0.05); & indicates a significant difference compared with the NaB+H2O2 group (*P* < 0.05).

As shown in Figures 7(a) and 7(b), compared with the NaB+H_2_O_2_ group, Mdivi-1 or Compound C treatment significantly reduced Beclin-1 levels (*P* < 0.05) and increased P62 levels (*P* < 0.05). Similarly, compared with the NaB+H_2_O_2_ group, Mdivi-1 or Compound C treatment also reduced the expression levels of mitophagy proteins PINK1 and Parkin (*P* < 0.05). Immunofluorescence result showed that inhibition of mitophagy or AMPK*α* reduced the colocalization of LC3B and mitochondria and reduced the colocalization of PINK1 and LC3B (Figures 7(c) and 7(d)). These results indicate that inhibition of mitophagy and AMPK*α* suppresses the induction of mitophagy by sodium butyrate.

### 3.8. The Effect of Sodium Butyrate on Mitophagy, Oxidative Stress, and Intestinal Epithelium Barrier of IPEC-J2 Cells after Interference with AMPK*α*

**Figure 8:**
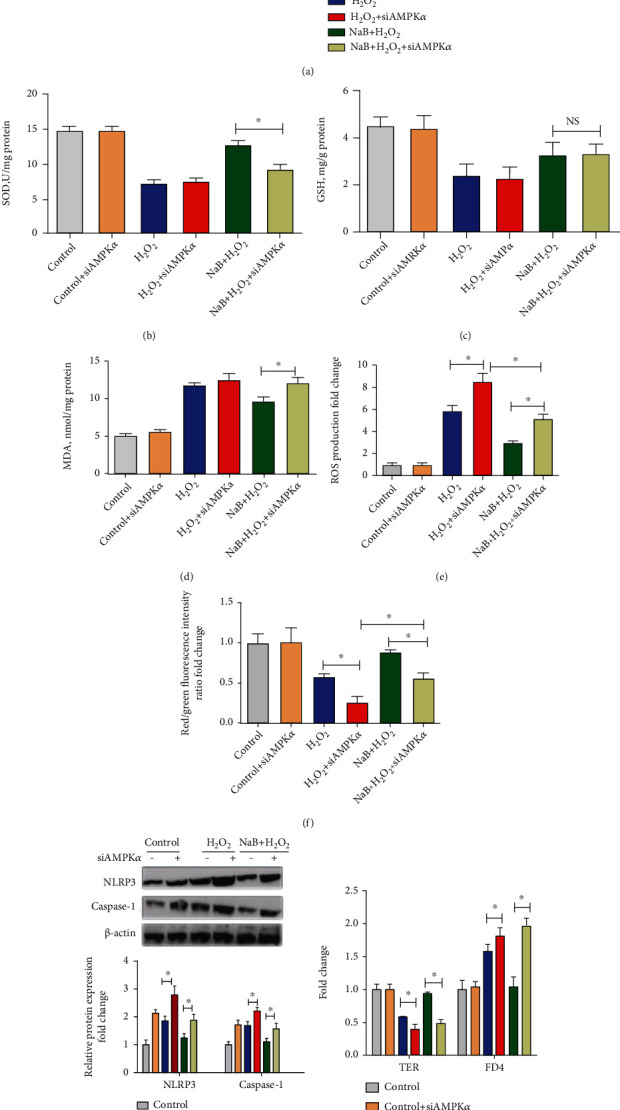
Effects of sodium butyrate (NaB) on mitophagy, oxidative stress, and intestinal epithelium barrier after interference with AMPK*α*. (a) Expression and quantification of mitophagy proteins PINK1, Parkin, and P62. (b–d) Superoxide dismutase (SOD), glutathione reductase (GSH) activity, and malondialdehyde (MDA) content of porcine intestinal epithelial cells (IPEC-J2). (e) Cellular reactive oxygen species (ROS) level of IPEC-J2. (f) Cellular mitochondrial membrane potential of IPEC-J2. (g) Protein expression and quantification of recombinant NLR family, pyrin domain-containing protein 3 (NLRP3) and Caspase-1. (h) Intestinal epithelial transepithelial resistance (TER) and fluorescein isothiocyanate dextran 4 kDa (FD4) permeability. ∗ indicates a significant difference compared with the control group (*P* < 0.05).

As shown in Figure 8(a), interference with AMPK*α* reduced (*P* < 0.05) the protein expression of PINK1 and Parkin and increased the protein expression level of P62 (*P* < 0.05) when compared with the H_2_O_2_ group and the NaB+H_2_O_2_ group. The results indicated that interference with AMPK*α* blocked sodium butyrate-induced enhancement of mitophagy. As shown in Figures 8(b)–8(f), the results showed that interference with AMPK*α* significantly reduced the activity of SOD and increased the level of MDA (*P* < 0.05) when compared with the NaB+H_2_O_2_ group. The interference with AMPK*α* increased the production of ROS (*P* < 0.05) and decreased the mitochondrial membrane potential (*P* < 0.05) when compared with the H_2_O_2_ group and the NaB+H_2_O_2_ group, indicating that the role of sodium butyrate on alleviating cellular oxidative stress and mitochondrial dysfunction was counteracted. From the results, it could be concluded that AMPK*α*-mediated mitophagy is necessary for sodium butyrate's protective effects on cellular redox status and mitochondrial function. As shown in Figures 8(g) and 8(h), interference with AMPK*α* significantly increased the protein expression of NLRP3 and Caspase-1 when compared with H_2_O_2_ and NaB+H_2_O_2_ groups (*P* < 0.05), indicating that interference with AMPK*α* significantly reduced the inhibition of NLRP3 activation by sodium butyrate. At the same time, interference with AMPK*α* resulted in a decrease in TER (*P* > 0.05) and an increase in FD4 (*P* > 0.05), indicating that interference with AMPK*α* weakened the protective effect of sodium butyrate on the intestinal epithelium barrier. From the results, it could be concluded that AMPK*α* is necessary for sodium butyrate to inhibit intestinal inflammation and protect intestinal epithelium barrier of IPEC-J2 cells under oxidative stress.

## 4. Discussion

Ma et al. reported that sodium butyrate could alleviate the intestinal epithelium barrier damage of IPEC-J2 cells and regulate cellular antioxidant level [8]. So far, no data was found on the effect of sodium butyrate on oxidative stress-induced intestine damage in IPEC-J2 cells. The present study demonstrated that sodium butyrate protected IPEC-J2 cells from H_2_O_2_-induced oxidative stress, as indicated by the increased SOD and GSH activities and the decreased MDA and ROS level in IPEC-J2 cells. Similarly, Russo et al. found that butyrate could effectively inhibit the decrease of glutathione transferase (GST) activity and the increase of ROS level induced by LPS in Caco-2 cells [27]. Excessive production of ROS would affect the electronic respiration chain of mitochondria, open mitochondrial permeability transition pores, and lead to depolarization of mitochondrial membranes [28]. We found that sodium butyrate alleviated H_2_O_2_-induced decrease in mitochondrial membrane potential (MMP), as indicated by the decreased proportion of depolarized cells and the increased ratio of red (JC-1 polymer)/green (JC-1 polymer) fluorescence intensity when compared with the H_2_O_2_ group. Consistent with our research, Li et al. reported that sodium butyrate inhibited LPS-induced MMP loss of bovine mammary epithelial cells [29]. Increased ROS production in mitochondria and decreased MMP in mitochondria can lead to mitochondrial DNA damage, which could be related to abnormal mitochondrial biogenesis [28]. We found that sodium butyrate alleviated mitochondrial dysfunction by increasing mtDNA and mRNA expression levels of mitochondrial function-related genes TFAM, NRF-1, and PGC-1*α*. Similarly, a previous study found that sodium butyrate alleviated the oxidative damage of HepG2 cells, as indicated by increasing mtDNA copy number and the mRNA expression of PGC-1*α* and TFAM [30]. Changes in mitochondrial function will be accompanied by changes in mitochondrial ultrastructure. Our research showed that H_2_O_2_ caused obvious mitochondrial swelling, respiratory ridge breakage, and mitochondrial vacuolation while sodium butyrate pretreatment significantly improved the ultrastructure of mitochondria. The changes in the ultrastructure of mitochondria are not only the structural basis of mitochondrial oxidative damage but also lead to the release of Cyt c from mitochondria into the cytoplasm. We found that H_2_O_2_ increased Cyt c level in the cytoplasm and decreased Cyt c level in mitochondria, suggesting that H_2_O_2_ increased the permeability of the inner and outer mitochondrial membranes, causing the release of mitochondrial Cyt c into the cytoplasm. Meanwhile, sodium butyrate alleviated the release of mitochondrial Cyt c induced by H_2_O_2_, indicating that sodium butyrate treatment could help improve mitochondrial function under oxidative stress. The above results revealed that sodium butyrate could effectively alleviate H_2_O_2_-induced redox status imbalance and mitochondrial impairment.

The intestinal barrier is an important line of defense for the body to prevent harmful substances from invading the body, and maintaining its integrity is vital to the health of the body [31, 32]. We found that sodium butyrate significantly alleviated H_2_O_2_-intestinal epithelium barrier injury of IPEC-J2 cells, as indicated by increasing intestinal epithelium TER and decreasing FD4 permeability. Similarly, Valenzano et al. and Wang et al. found that treatment of Caco-2 cells with a certain concentration of butyric acid could effectively improve the intestinal epithelium barrier [33, 34]. Sodium butyrate also significantly inhibited the decrease of Claudin-1, Occludin, and ZO-1 in IPEC-J2 cells induced by H_2_O_2_. Consistent with our research, Yan and Ajuwon found that sodium butyrate increased the protein level of Claudin-3 and Claudin-4, thereby alleviating the damage of LPS to the integrity of IPEC-J2 monolayer cells [35]. The integrity of intestinal epithelial cells is not only related to the expression level of tight junction protein but also affected by its distribution area. In our study, we found that after H_2_O_2_ treatment, the normal distribution and expression of Claudin-1 were destroyed and sodium butyrate prevented the disorder of Claudin-1 distribution and the decrease of Claudin-1 expression caused by H_2_O_2_. Similarly, studies found that when Caco-2 cells were adversely stimulated, the Occludin and ZO-1 proteins originally expressed on the cell membrane would be transferred to the cytoplasm, thereby increasing cell permeability; however, butyric acid promoted tight junction protein redistribute and increased the expression level of tight junction [9, 36]. These results indicated that sodium butyrate could effectively alleviate the intestinal epithelium barrier damage and the disorder of tight junction protein expression and distribution induced by H_2_O_2_ in IPEC-J2 cells.

Oxidative stress could cause intestinal inflammation, as proved by that excessive ROS production in the intestines of patients with ulcerative colitis and Crohn disease weakened antioxidant capacity and aggravated oxidative damage [27]. According to our results, H_2_O_2_ increased the mRNA and protein expression of NLRP3, Caspase-1, and IL-1*β* in IPEC-J2 cells and increased the content of IL-1*β*, while sodium butyrate pretreatment alleviated this phenomenon. Similarly, NLRP3 inflammasome and IL-1*β* were activated by H_2_O_2_ in rat livers [37]. Jiang et al. also found that in bovine macrophages, sodium butyrate could reduce the inflammatory response caused by LPS by inhibiting the NF-*κ*B and NLRP3 signaling pathways [38]. According to the results, we assumed that sodium butyrate could effectively inhibit the activation of NLRP3 and relieve intestinal inflammation induced by H_2_O_2_.

Mitophagy is a self-protection mechanism to remove dysfunctional mitochondria [39]. However, there were no reports about the effect of sodium butyrate on mitophagy of IPEC-J2 cells. We found that sodium butyrate enhanced mitophagy of IPEC-J2 cells, as indicated by increasing mRNA and protein level of mitophagy protein and promoting the colocalization of LC3B and mitochondria as well as the colocalization of PINK1, Parkin, and LC3B; meanwhile, we also found that sodium butyrate increased mitophagy vesicles under TEM. Similarly, Zhou et al. found that butyric acid can regulate intestinal epithelial cell autophagy through the HIF-1*α* pathway to alleviate colitis [40]. Wang et al. found that sodium butyrate activated the PINK1-Parkin pathway and induced mitophagy [41]. J. S. Lee and G. M. Lee also found that sodium butyrate could induce mitophagy in Chinese hamster ovary (CHO) cells, and mitophagy protein Parkin was recruited to mitochondria, suggesting that sodium butyrate induced mitophagy to remove damaged mitochondria [14]. According to the results above, it was reasonable to assume that sodium butyrate could regulate PINK1 and Parkin and trigger mitophagy to obliterate the damaged mitochondria caused by H_2_O_2_, which could prevent the intestine from metabolism disorders.

In the present experiment, we found that sodium butyrate protected IPEC-J2 cells from H_2_O_2_-induced oxidative damage, intestinal epithelium barrier damage, and mitochondrial impairment and enhanced mitophagy, but its specific molecular mechanism remained to be further studied. AMPK is a key energy sensor that can regulate cell energy metabolism [15]. Mollica et al. have reported that feeding sodium butyrate to insulin-resistant obese mice can increase liver AMPK activity, reduce ROS production, and improve liver mitochondrial function [20]. However, whether sodium butyrate could alleviate oxidative stress of IPEC-J2 cells through AMPK-dependent mitophagy remained to be further explored. Therefore, in the following experiments, we used AMPK inhibitor and mitophagy inhibitor to explore the specific role of AMPK signaling pathway and mitophagy in the protective effects of sodium butyrate on intestinal epithelium barrier. We found that sodium butyrate inhibited the decrease of SOD activity and the increase of MDA level induced by H_2_O_2_. However, the use of mitophagy inhibitor Mdivi-1 or AMPK inhibitor Compound C increased cellular MDA levels and reduced SOD activity. These results indicated that inhibition of mitophagy and AMPK could reduce the antioxidant effect of sodium butyrate. Similarly, Guo et al. found that butyric acid activated the AMPK signaling pathway through GPR109A in bovine mammary epithelial cells (BMECs) and then exerted an antioxidant effect [37]. We found that the ROS level and the percentage of depolarized cells significantly increased after using the mitophagy inhibitor Mdivi-1 or the AMPK inhibitor Compound C. Similarly, Zhao et al. reported that high concentrations of insulin in HepG2 cells could significantly reduce mitochondrial DNA and decrease the mitochondrial membrane potential, while sodium butyrate significantly increased the mitochondrial membrane potential and mitochondrial DNA, which was involved in the GPR43-*β*-AMPK signaling pathway [42]. We also previously reported that tributyrin activated the AMPK-mTOR signaling pathway and improved intestinal mitochondrial function in weaned piglets [25]. These results indicated that inhibition of mitophagy or AMPK*α* inhibited the protective effects of sodium butyrate on improving mitochondria under oxidative stress of IPEC-J2 cells. In addition, we found that the use of mitophagy inhibitor Mdivi-1 and AMPK inhibitor Compound C impaired intestinal epithelium barrier function, as indicated by the decreased TER and expression level of tight junction and the increased FD4 permeability of IPEC-J2 cells. Similarly, Elamin et al. reported that sodium butyrate alleviated the barrier dysfunction of Caco-2 cells induced by ethanol in vitro, of which the protective effects were suppressed by AMPK inhibitors or siRNA. Miao et al. found that in the Caco-2 cells, sodium butyrate activated AMPK, thereby promoting the reorganization of tight junctions [43]. Therefore, it was reasonable to assume that mitophagy and AMPK were necessary for sodium butyrate to improve the intestinal epithelium barrier under H_2_O_2_-induced oxidative stress. Moreover, we also found that the use of mitophagy inhibitor Mdivi-1 or AMPK inhibitor Compound C inhibited mitophagy, as indicated by decreasing expression level of mitophagy protein and the colocalization of PINK1 and LC3B, suggesting that inhibition of mitophagy or AMPK could suppress the mitophagy induced by sodium butyrate. In accordance with our results, Evans et al. found that butyrate induced autophagy-dependent cell apoptosis of human gingival epithelial cell Ca9-22 to alleviate periodontal disease, as indicated by activating AMPK and inducing the production of LC3B, which was attenuated by AMPK inhibition; in addition, interference with LC3B gene could also significantly inhibit butyrate-induced cell death [44]. Hence, AMPK-mediated mitophagy is necessary for sodium butyrate's protective effects against oxidative stress.

In order to further confirm the role of AMPK in sodium butyrate-induced mitophagy of IPEC-J2 cells, we then used siRNA technology to knock down AMPK*α*, which was known as the dominating AMPK catalytic subunit. We found that interference with AMPK*α* reduced the expression level of PINK1 and Parkin and increased the level of P62 when compared with the H_2_O_2_ group and the NaB+H_2_O_2_ group. Similarly, Luo et al. found that sodium butyrate increased the mRNA and protein expression level of LC3B and activated phosphorylated AMPK, which was suppressed by treatment with siRNA AMPK in colorectal cancer cells [45]. These results indicated that AMPK*α* can mediate the mitophagy induced by sodium butyrate. Next, we found that interference with AMPK*α* significantly increased the level of MDA and ROS production and decreased MMP, indicating that the protective effects of sodium butyrate on alleviating cellular oxidative stress and mitochondrial dysfunction were weakened. In addition, interference with AMPK*α* significantly increased the protein expression of NLRP3 and Caspase-1 in IPEC-J2 cells, indicating that interference with AMPK*α* weakened the inhibition effect of sodium butyrate on NLRP3 inflammation factors. At the same time, interference with AMPK*α* impaired intestinal epithelium barrier function, as indicated by decreasing TER and increasing FD4 permeability of IPEC-J2 cells, suggesting that interference AMPK*α* weakened the protective effects of sodium butyrate on the intestinal epithelium barrier. Our results suggested that sodium butyrate could promote mitophagy via AMPK activation, protect mitochondria, and exert a protective effect on intestinal epithelium barrier of IPEC-J2 cells under oxidative stress.

## 5. Conclusion

In conclusion, our work revealed that sodium butyrate ameliorated oxidative stress and inflammation and enhanced intestinal epithelium barrier function and mitochondrial function through AMPK-mitophagy pathway in IPEC-J2 cells.

## Data Availability

The data used to support the findings of this study are available from the corresponding author upon logical request.

## Abbreviations

AMPK: Adenosine 5′-monophosphate- (AMP-) activated protein kinase
CC: Compound C
Cyt c: Cytochrome c
FD4: Fluorescein isothiocyanate dextran 4 kDa
GSH: Glutathione reductase
H_2_O_2_: Hydrogen peroxide
IPEC-J2: Porcine intestinal epithelial cells
IL-1*β*: Interleukin-1*β*
SOD: Superoxide dismutase
LC3B: Light-chain-associated protein B
mtDNA: Mitochondrial DNA
MDA: Malondialdehyde
Mdivi-1: Mitochondrial division inhibitor 1
NRF-1: Nuclear respiratory factor-1
NaB: Sodium butyrate
NLRP3: Recombinant NLR family, pyrin domain-containing protein 3
PINK1: PTEN-induced putative kinase 1
PGC-1*α*: Peroxisomal proliferator-activated receptor-g coactivator-1*α*
ROS: Reactive oxygen species
TFAM: Mitochondrial transcription factor A
TER: Transepithelial resistance
ZO-1: Zonula occludens-1.

## Supplementary Materials

Supplementary Materials Table 1: primer sequence for different genes of quantitative real-time (RT-qPCR) analysis.
