# Supplementary material for: Sodium Butyrate Ameliorates Oxidative Stress-Induced Intestinal Epithelium Barrier Injury and Mitochondrial Damage through AMPK-Mitophagy Pathway
Source: Oxid Med Cell Longev. 2022 Jan 29;2022:3745135. doi: 10.1155/2022/3745135 (PMC8817854; doi:10.1155/2022/3745135)
Supplement: Supplementary Materials — Table 1: primer sequence for different genes of quantitative real-time (RT-qPCR) analysis. [file 3745135.f1.docx]

Table S1. Primer sequence for different genes

| Primer name | Forward (5′-3′) | Reverse (5′-3′) |
| --- | --- | --- |
| ASC | GACAACAAACCAGCACTGCAC | CCTCCTCATTTTGGTGGGGT |
| ATG5 | CCCTCTTGGGGTACATGTCT | CGTCCAAACCACACATCTCG |
| Beclin-1 | AGGAGCTGCCGTTGTACTGT | CACTGCCTCCTGTGTCTTCA |
| Caspase-1 | ATCTCACCGCTTCGGACATGGCTA | GTATTTCTTCCCACAAATGCCAGCC |
| GAPDH | ACACTCACTCTTCTACCTTTG | GAAATTCATTGTCGTACCAG |
| IL-1β | CAAAGGCCGCCAAGATATAA | GAAATTCAGGCAGCAACAT |
| LC3 | CCGAACCTTCGAACAGAGAG | AGGCTTGGTTAGCATTGAGC |
| NLRP3 | CCTTCAGGCTGATTCAGGAG | GACTCTTGCCGCTATCCATC |
| NRF-1 | GCCAGTGAGATGAAGAGAAACG | CTACAGCAGGGACCAAAGTTCAC |
| P62 | AGTGTCCGTGTTTCACCTTCC | TGCCCAGACTACGACTTGTG |
| PINK1 | CCCCTCACCCCAACATCATC | AGCACATCGGGGTAGTCAAC |
| PARKIN | AACTCCAGCCATGGTTTCCC | CGCTTAGCAACCACCTCCTT |
| PGC-1α | CCCGAAACAGTAGCAGAGACAAG | CTGGGGTCAGAGGAAGAGATAAAG |
| TFAM | GGTCCATCACAGGTAAAGCTGAA | ATAAGATCGTTTCGCCCAACTTC |
